# Senescence-Associated Secretory Phenotype Determines Survival and Therapeutic Response in Cervical Cancer

**DOI:** 10.3390/cancers12102899

**Published:** 2020-10-09

**Authors:** Sharad Purohit, Wenbo Zhi, Daron G. Ferris, Manual Alverez, Lynn Kim Hoang Tran, Paul Minh Huy Tran, Boying Dun, Diane Hopkins, Bruno dos Santos, Sharad Ghamande, Jin-Xiong She

**Affiliations:** 1 Center for Biotechnology and Genomic Medicine, Medical College of Georgia, Augusta University, 1120, 15th St, Augusta, GA 30912, USA; spurohit@augusta.edu (S.P.); wzhi@augusta.edu (W.Z.); lytran@augusta.edu (L.K.H.T.); ptran@augusta.edu (P.M.H.T.); bdun@augusta.edu (B.D.); dhopkins@augusta.edu (D.H.); 2 Department of Obstetrics and Gynecology, Medical College of Georgia, Augusta University, 1120, 15th St, Augusta, GA 30912, USA; dferris@augusta.edu (D.G.F.); sghamande@augusta.edu (S.G.); 3 Department of Undergraduate Health Professionals, College of Allied Health Sciences, Augusta University, 1120, 15th St, Augusta, GA 30912, USA; 4 Department of Gynecologic Oncology, Instituto Nacional de Enfermedades Neoplasicas, Surquillo, Lima 15038, Peru; malvarez@inen.sld.pe; 5 Jinfiniti Precision Medicine, Inc., Augusta, GA 30912, USA; BDOSSANTOS@augusta.edu

**Keywords:** gynecologic cancers, cervical neoplasia, serum proteins, proteomics, biomarkers, radiation therapy, prognosis

## Abstract

**Simple Summary:**

Cervical cancer is the most common gynecological cancer caused by persistent infections with human papilloma viruses. Over time, this infection leads to secretion of inflammatory proteins in the cervix, which exacerbates the neoplastic and senescent changes to the cervical epithelial lining. We measured nineteen serum proteins in retrospectively collected samples from cervical cancer patients. We show here that 10 out of 19 proteins are associated with senescence phenotype in cervical cancer patients. This senescence associated protein signature influences how cervical cancer patients responds to therapy.

**Abstract:**

Molecular biomarkers that can predict survival and therapeutic outcome are still lacking for cervical cancer. Here we measured a panel of 19 serum proteins in sera from 565 patients with stage II or III cervical cancer and identified 10 proteins that have an impact on disease specific survival (DSS) (Hazzard’s ratio; HR = 1.51–2.1). Surprisingly, all ten proteins are implicated in senescence-associated secreted phenotype (SASP), a hallmark of cellular senescence. Machine learning using Ridge regression of these SASP proteins can robustly stratify patients with high SASP, which is associated with poor survival, and patients with low SASP associated with good survival (HR = 3.09–4.52). Furthermore, brachytherapy, an effective therapy for cervical cancer, greatly improves survival in SASP-high patients (HR = 3.3, *p* < 5 × 10^−5^) but has little impact on survival of SASP-low patients (HR = 1.5, *p* = 0.31). These results demonstrate that cellular senescence is a major determining factor for survival and therapeutic response in cervical cancer and suggest that senescence reduction therapy may be an efficacious strategy to improve the therapeutic outcome of cervical cancer.

## 1. Introduction

Cervical cancer is the most common gynecological cancer, responsible for an estimated 311,365 deaths worldwide [1]. In 99% of cervical cancer cases, infections by human papilloma viruses (HPV) [2] is the causative agent; however, the majority of the infections do not progress to cancer. Persistent infections with high-risk HPV leads to integration of E6 and E7 oncogenes into the host genome [3]. In the early phase, the E6 and E7 oncogenes-encoded proteins target the tumor suppressor genes such as p53 and Rb [4], and also play a role in altering immune response by deregulating the JAK-STAT pathway [3].

Once established the HPV promotes alterations in the immune system by secretion of inflammatory cytokines and immune cell infiltrations in cervix [5]. In a recent study increased levels of inflammatory cytokines were observed in women <50 years of age with cervical intraepithelial neoplasia (CIN) and invasive cervical carcinoma (ICC) [6]. Sustained release of the cytokines and inflammatory mediators by the neoplastic cells of the cervix leads to migration of immune cells into the cervical microenvironment, which has been shown to exacerbate the neoplastic changes [7]. Increased infiltration of immune cells have been observed in women <50 years of age reported to clinic with high-grade squamous intra-epithelial lesions and ICC [8]. Elevated levels of circulating cytokines can distinguish low- and high-grade lesions from ICC [6,8]. The sustained elevation in inflammation contributes to the HPV-mediated tumorigenesis by production of reactive oxygen species (ROS), activation of inflammatory pathways leading to increased cell proliferation and senescence [5,7,9]. The continuous proliferation and senescence lead to DNA damage that leads to neoplastic changes in the cervix [10].

In this study, we measured serum levels of 19 proteins using Luminex multiple protein array to assess their potential role in determining the therapeutic outcome in a large cohort of Peruvian cervical cancer patients. We report that nine serum proteins have a substantial impact on the survival of squamous cell cancer of the cervix (SCCC). All nine proteins are part of the senescence-associated secreted phenotype (SASP), suggesting that cellular senescence is a key determining factor for therapeutic response and survival for SCCC.

## 2. Results

### 2.1. Patient Characteristics 

The clinical and demographic data on squamous cell carcinoma of cervix patients (SCCC, *n* = 565) with survival data is present in Table 1. 

The median age of all study subjects was 49 years (range 26 to 82 years). The study subjects were grouped into three datasets based on the stage and treatment regimen. All stage II patients (*n* = 276) received external beam radiation therapy (EBRT) plus brachytherapy (BT) and will be referred to as RTBT2 group/dataset. Most stage III patients (*n* = 203) also received EBRT plus BT and will be referred to as RTBT3 group, while 86 stage III patients only received EBRT and did not receive brachytherapy (referred to as RT3 group). As we have shown previously [11], RTBT2 patients have better disease specific survival (DSS) than RTBT3 patients (hazards ratio; HR = 2.3), indicating a significant influence of stage on DSS. Furthermore, RTBT3 patients have much better survival than RT3 patients (HR = 6.7), suggesting that brachytherapy may be associated with substantially better outcome (Table 1). 

### 2.2. Individual Proteins Associated with SCCC Survival

We tested 19 proteins using Luminex multiplex bead array. The dataset was divided into 4 quartiles containing 25% of the study subjects. We used serum levels in Quartile 1 as a reference to compare levels in quartile 2–4 (Q2–Q4). The hazard ratios (HR) observed for individual proteins in 3rd quartile showed elevated protein levels compared to Q1 subjects but there is no statistically significant difference. Seven proteins showed significant associations between serum levels in 4th quartile (Q4) and poor DSS were, CRP (Quartile 4 (Q4) HR: 1.89; 95% CI:1.27–2.81; *p* = 0.00162), GRO-α (HR: 2.07; 95% CI:1.4–3.07; *p* = 0.00027), HGF (HR: 1.78; 95% CI: 1.18–2.69; *p* = 0063), MIG (HR: 1.85; 95% CI: 1.23–2.77; *p* = 0.0028), MMP1 (HR: 1.69; 95% CI: 1.28–2.26; *p* = 2.6 × 10^−4^), SAA (HR: 1.6; 95% CI: 1.19–2.16; *p* = 0.0021), and PAI-1 (HR: 1.79; 95% CI: 1.22–2.62; *p* = 0.0026) (Table 2). While higher leptin levels (Q4 HR: 0.61; 95% CI: 0.41–0.90; *p* = 0.014) were found to be associated with better DSS. Statistical evidence for six proteins including GRO, CRP, HGF, MIG, MMP1 and PAI-1 is strong and still significant after correcting for multiple testing. 

### 2.3. Subgroup Analysis for Individual Proteins

**Figure 1:**
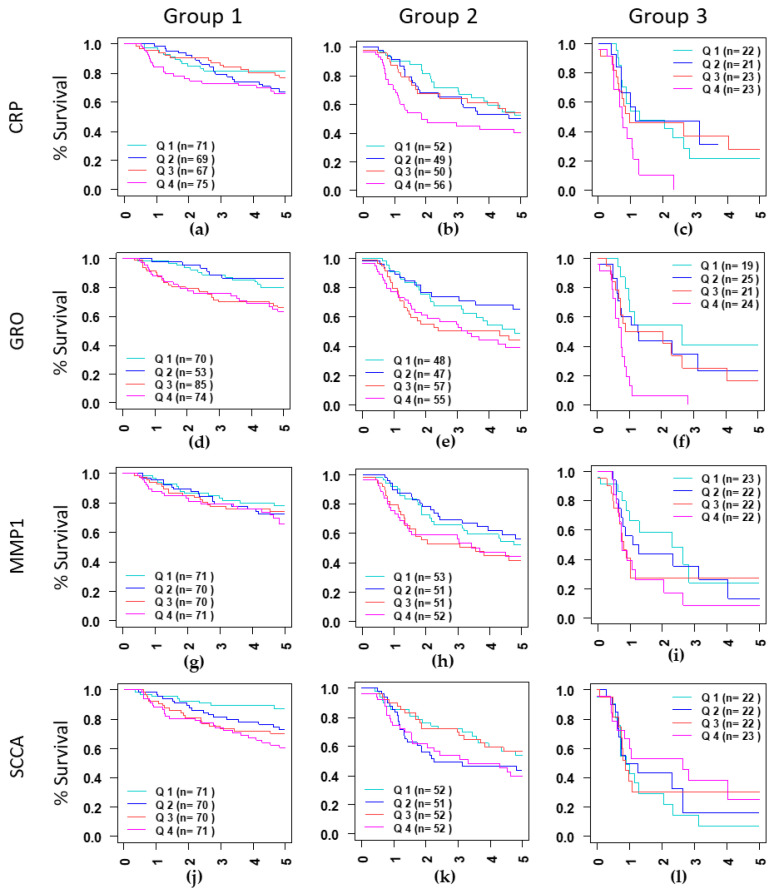
Kaplan-Meir curves curves for CPR (**a**–**c**), showing differences in survival in three groups identified based on stage (II and III) and treatment type (EBRT and EBRT+BT). Group 1: Stage II treated with EBRT+BT (**a**,**d**,**g**,**j**), Group 2: Stage III treated with EBRT+BT (**b**,**e**,**h**,**k**) and Group 3: Stage III treated with EBRT alone (**c**,**f**,**i**,**l**). serum level for each protein was divided into quartiles containing 25% of patients, Q1: Quartile 1 (0–25%), Q2: Quartile 2 (25–50%), Q3: Quartile 3 (50–75%) and Q4: Quartile 4 (75–100%). Quartile 1 was compared to Q2–Q3.

Since DSS differs significantly by stage and treatment type and significant interactions between treatment and protein were seen for some proteins such as PAI-1, group-specific analyses were conducted in the three patient groups that are homogeneous for stage and treatment. In general, the significant proteins identified in the entire dataset are also significantly associated with survival in the RTBT2 and RT3 datasets; however, fewer significant proteins were observed in the RTBT3 dataset (Table 3). Notable exceptions include SCCA, sIL2Rα, and PAI-1. Elevated SCCA (Q4) is associated with poor survival in RTBT2 (HR = 3.55, *p* = 0.0018) but not in RTBT3 and RT3. Similarly, increased sIL2Rα (both Q4 and Q3) is marginally associated with poor survival in RTBT2 patients but not in RTBT3 and RT3 datasets. These associations were not revealed in the analyses of the entire cohort, probably due to differences between the datasets. In contrast, elevated PAI-1 (Q4) is strongly associated with poor survival (HR = 3.98, *p* = 0.0007) in the RT3 dataset but not in the RTBT2 and RTBT3 datasets, probably reflecting a difference related to treatment modalities. Kaplan-Meir survival curves for the selective proteins are shown in Figure 1.

### 2.4. Multi-Protein Models Have Greater Prognostic Potential for RTBT2 Patients

Surprisingly, all proteins significantly associated with SCCC survival are implicated in cellular senescence and are either senescence-associated secretory phenotype (SASP) proteins or are involved in the regulation of SASP (see discussion). Therefore, we attempted to develop SASP models/scores and evaluated their potential utility as SCCC prognosis biomarkers. Our analytical pipeline includes: (1) computation of linear predictor values for each patient using Ridge regression for multiple proteins, (2) assign each patient into two or more subgroups using the linear predictor values, and (3) evaluation of survival of the subgroups using Cox proportional analysis. In contrast to the conventional approach that develops one Ridge model using all patients in the dataset, we sampled 3000 training and test pairs, each containing 50% of the patients in the dataset. This procedure allowed us to generate and test 1000 different models. Additional models may be generated and tested if desired. This analytical pipeline was first applied to the RTBT2 dataset with a panel of 8 proteins (CRP, GRO, LEPTIN, MIG, MMP1, SCCA SAA, and sIL2Rα) that showed significant association with survival at the individual protein level. The sampled training and testing datasets were divided into SASP-low and SASP-high subset using 40th percentile as cutoff value. The analyses generated a number of models with consistent results in the training and testing pairs (HR for high SASP > 3.5), suggesting that the multi-protein models have much better prognostic value than any individual proteins. Data for the top 17 models are summarized in Table 3. 

**Figure 2:**
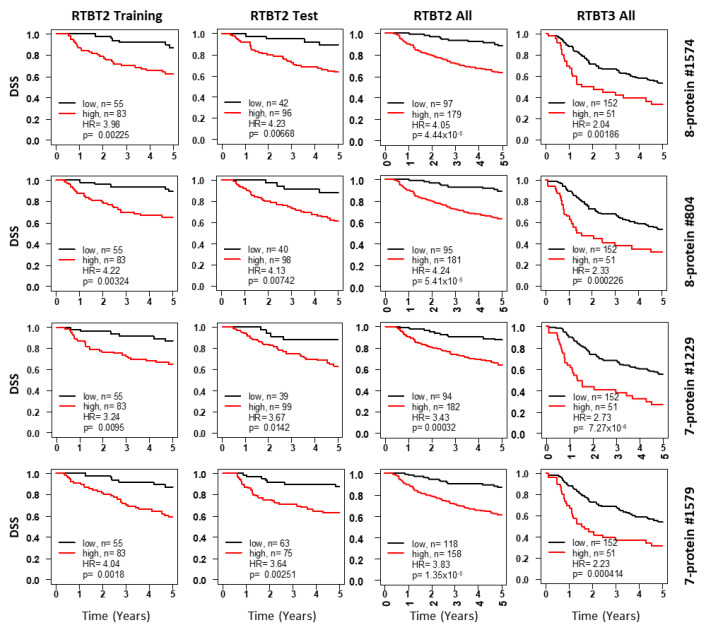
Kaplan-Meir curves for Ridge models developed with the RTBT2 dataset. Shown for each representative model are survival curves for the training subset (column 1), testing subset (column2), all RTBT2 dataset (column3) and validation in the RTBT3 dataset (column4). Patients in each training subset were divided into high (60%) versus low (40%) for survival comparison and the cutoff threshold was applied to the testing and the independent RTBT3 dataset.

Furthermore, we also generated and tested RTBT2 models in a similar way using seven proteins (CRP, GRO, LEPTIN, MIG, MMP1, SCCA and HGF), of which six are also included in the 8-protein models. This panel of seven proteins yielded 14 excellent models with consistent HRs in the training and test pairs (Table 3). Kaplan-Meir survival curves for the selective models shown in Figure 2 confirm the excellent prognostic potential for these multi-protein models.

The robustness of these top models identified by the training and testing pairs were further evaluated by 1000 iterations of bootstrapping. Each bootstrap sampled 70% of the RTBT2 patients and the bootstrapped patient subsets were divided into two subsets at the 40th percentile cutoff based on the Ridge regression score of each model. HR and p value were computed for each bootstraped data set. Data for each model are summarized in Table 3, which shows the mean HR for 1000 bootstraps and the number of bootstraps with different levels of p value. Conventionally, the models are validated if 95% of the models have p values of <0.05. All 31RTBT2 models showed high robustness with >98.5% of the bootstraps having *p* < 0.05. The mean HR of these bootstraps range from 3.13 to 4.52, suggesting that these models are robust and can reliably identify patients with high SASP and bad prognosis.

These 31 RTBT2 models were further validated in the independent RTBT3 dataset to determine their performance on a dataset that contains patients with a higher stage but received the same therapy as RTBT2. As shown in Table 3, all 31 RTBT2 models were confirmed to be able to identify RTBT3 patients with high SASP score and worse survival, providing further evidence that the 31 RTBT2 models are good biomarkers for both stage II and stage III patients. 

### 2.5. SASP Models Optimized for RT3 Patients

**Figure 3:**
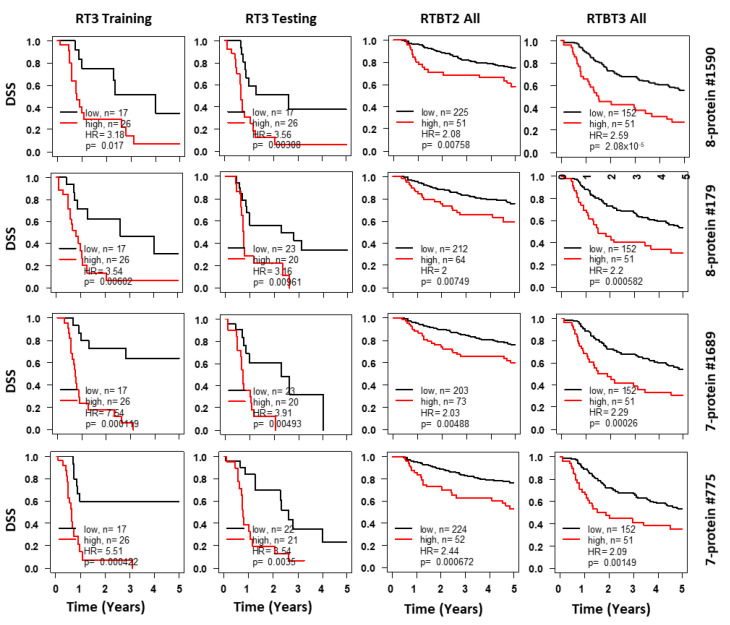
Kaplan-Meir curves for Ridge models developed with the RT3 dataset. Shown for each representative model are survival curves for the training subset (column 1), testing subset (column 2), validation in the RTBT2 (column 3) and RTBT3 datasets (column 4). Patients in each training subset were divided into high (40%) versus low (60%) for survival comparison and the cutoff threshold was applied to the testing and the independent RTBT2 and RTBT3 datasets. Results for selected protein models are presented here.

The models derived from the RTBT2 dataset were not expected to perform very well for the RT3 patients who differ from RTBT2 by both stage and treatment modality. Therefore, we searched new models for RT3 patients by applying the analytical pipeline to the RT3 dataset using the same eight and seven protein sets. The top 25 selected models have HRs between 3.05 and 3.93 in training and between 3.04 and 5.23 in testing (Table 4 and Figure 3). All 25 models were validated by 1000 iterations of bootstrapping and the mean HRs from bootstrapping were also high (HR = 2.93–4.61) (Table 4). All 25 models except one were also validated in the RTBT3 dataset and all models were validated in RTBT2 dataset (Table 4).

### 2.6. Model Consistency and Plurality Voting (Consensus Model)

**Figure 4:**
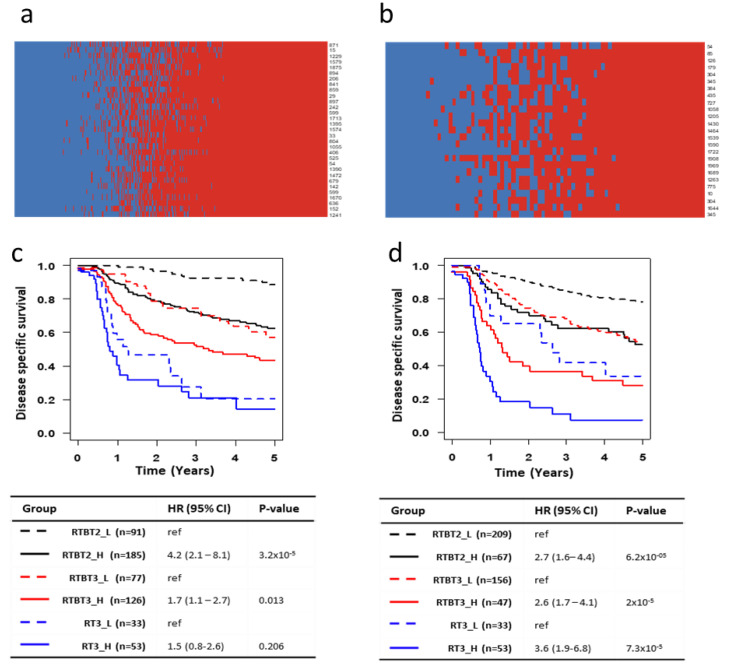
Model consistency and plurality voting for patient classification. (**a**,**b**) Heatmaps showing the voting by each model (row) on each patient (column). Red: SASP_H; Blue: SASP_L. Voting results are shown for all RTBT2 patients by RTBT2 models (**a**) and RT# patients by RT3 models (**b**). (**c**) Kaplan-Meir survival curves for SASP_H and SASP_L subsets in each of the three datasets (RTBT2, RTBT3 and RT3). SASP groups were defined by plurality voting of all 31 RTBT3 Ridge models. A patient is considered as SASP_H if >50% of the models voted the patient as SASP_H. (**d**) Kaplan-Meir survival curves for SASP_H and SASP_L subsets in each of the three datasets (RTBT2, RTBT3 and RT3). SASP groups were defined by plurality voting of all 25 RT3 Ridge models. A patient is considered as SASP_H if >50% of the models voted the patient as SASP_H.

Because multiple models can potentially predict survival, it is essential to determine how consistently these different models classify each patient. Model consistency would be further evidence that the identified models are valid. Each model was used to assign each patient to either SASP-H or SASP-L group. The classification data for RTBT2 models on RTBT2 patients are summarized by the heatmap in Figure 4a, while the RTBT3 models on RT3 patients are summarized in Figure 4b. 

The final classification of a patient is determined by plurality voting of all models and the confidence of the classification is for each patient can be assessed by the percentage of models voting the patient into a SASP group. For the RTBT2 group 74.9% of patients were classified with 75–100% confidence (most with 100% confidence), 25.1% of the patients have lower confidence and are referred to as medium SASP (SASP_M). Similarly for RT3 patients, 79.1% are classified with 75–100% confidence while 20.9% of the patients are classified with lower confidence. These results suggest that these models classify are largely consistent. The consensus model identifies patients with significant survival differences comparable or better than individual models (Figure 4c,d). The survival difference between SASP_H and SASP_L patients is quite dramatic. For example, the RT3_H patients have a one year survival of about 20% and a five year survival of about 10% compared to one year survival of 70–90% and five year survival of 40–60% for the RT3-L patients.

### 2.7. Multifactorial Prognostication by Stage, Treatment and SASP

Our previous study has shown that stage and especially treatment type are major determinants of SCCC survival in this cohort [11]. Figure 4c,d show the collective influence of stage, treatment and SASP score on survival. Stage 2 SASP_L patients treated with brachytherapy (RTBT2_L group) have the best prognosis (five year survival of about 80%), while stage 3 SASP_H patients without brachytherapy (RT3_H) have the worst prognosis (5-yrs survival of about 10%). The next best survival rate is observed in stage 2 SASP_H patients (RTBT2_H) and stage 3 SASP_L patients (RTBT3_L) who received brachytherapy (5-yrs survival of about 50%).

### 2.8. SASP Has a Major Impact on Response to Brachytherapy

Brachytherapy is known to provide benefit to SCCC patients and was shown to be associated with much better survival in this data set (HR = 6.7) [11,12]. Here, we explored whether brachytherapy improves survival for all stage 3 patients or only a subset of stage 3 patients. 

**Figure 5:**
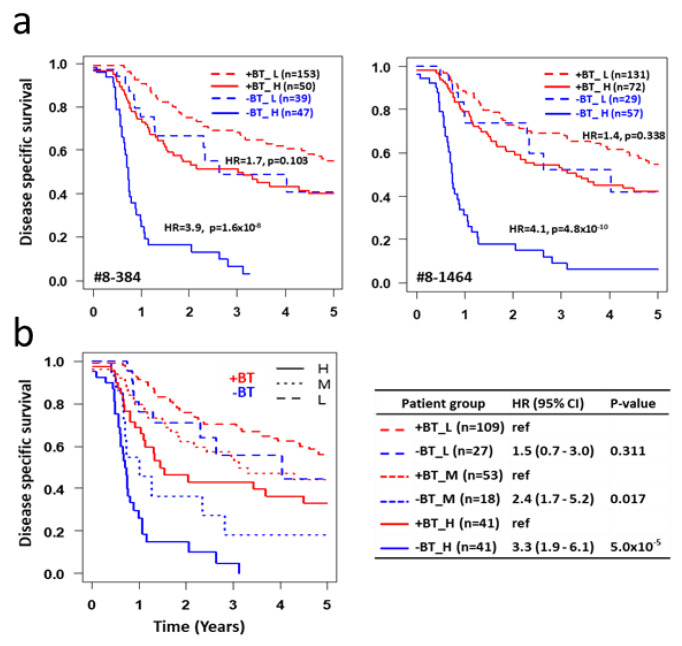
Impact of SASP on response to brachytherapy. (**a**) All stage 3 patients (RTBT3+RT3) were classified into four subsets based on brachytherapy status (+BT and -BT) and SASP status (H vs. L) using cutoffs for each of the 25 RT3 models. Survival curves are shown for all four subsets for two representative models. HR and p values are shown between +BT and -BT subsets within SASP_H or SASP_L subsets. Data for all 25 models are shown in Table 3. (**b**) All stage 3 patients are classified into four subsets based on brachytherapy status and SASP status using plurality voting of all 25 RT3 models. SASP_H: >75% models voted the patient as SASP_H; SASP_L: >75% models voted the patient as SASP_L; SASP_M: <75% of models voted the patient as SASP_H or SASP_L. Data is summarized on the right.

To answer this question, all stage 3 patients (RTBT3 and RT3) are combined into one dataset and classified SASP_L or SASP_H group based on their SASP score from each of the 25 RT3 models. Survival was assessed between brachytherapy and no brachytherapy within SASP_L and SASP_H patient groups. Surprisingly, irrespective of the 25 models examined, brachytherapy has no significant impact on survival for SASP_L patients while brachytherapy significantly improves survival of SASP_H patients (HR > 4.0 for the best models) (Figure 5a, Table 5).

Using plurality voting of the 25 RT3 models, all stage 3 patients were classified into three SASP groups: H (>75% models voting high), L (>75% models voting low) and M (less 75% models voting either high or low). Consistent with the data from individual models, brachytherapy does not provide a significant survival benefit for SASP_L patients (HR = 1.5, *p* = 0.311), which represent 47% of all stage 3 patients, while brachytherapy does provide a significant survival benefit to SASP_H patients (HR = 3.3, *p* < 5 × 10^−5^) and SASP_M patients (HR = 2.4, *p* = 0.017) (Figure 5b).

## 3. Discussion

This study profiled 19 serum proteins in 565 SCCC patients categorized in three phenotypic groups based on disease stage and treatment modalities, both having a major impact on DSS. Statistical analyses of single proteins revealed 10 proteins that are significantly associated with DSS in at least one of the patient groups. All these proteins, with the exception of leptin, are elevated in patients with poor survival in the stage- and treatment-matched patient groups. Surprisingly, every single protein is part of the senescence-associated secretory phenotype (SASP) or is implicated in the regulation of SASP, which is a hallmark of cellular senescence, a programmed cell response leading to a permanent cell cycle arrest. Cellular senescence is a tumor-suppressive mechanism that permanently arrests cells at risk of malignant transformation on one hand, but SASP turns senescent cells into pro-inflammatory cells with the ability to promote tumor progression [13]. Multiple prognostic proteins identified in this study are part of SASP, including MMP1, growth-related oncogene (GRO), encoded by the *CXCL1* gene, monokine induced by IFNγ (MIG), encoded by the *CXCL9* gene. These pro-inflammatory chemokines are responsible for excessive neutrophil recruitment to the site of inflammation [14]. Neutrophils produce ROS and secrete other pro-inflammatory molecules for recruitment of macrophages and T-cells [15], driving the neoplastic processes.

IL2Rα (CD25) is increased in senescent T cells [16] and thus component of SASP. It has been reported that the soluble sIL2Rα, an antagonist of IL2 signaling, is elevated in response to disease severity in cervical cancer patients [17], whereas IL2 levels declined with disease severity [18,19]. The sIL2Rα is generated by proteolytic cleavage from the cell surface of activated T and NK cells, monocytes and tumor cells and is shown to play a role in cancer mediated immune suppression [20]. The poor prognosis in cervical cancer patients with elevated IL2Rα as part of the senescence phenotype may be explained by a lack of Th1 response as the consequence of immune suppression through sequestering free IL2 [17,20,21].

Plasminogen activator inhibitor-1 (PAI-1), a member of the evolutionarily conserved serine protease inhibitor family, is a potent and rapid-acting inhibitor of the mammalian plasminogen activators. Increased PAI-1 production guides the onset and progression of a number of human diseases and contributes to the age-related morbidities. Cellular senescence, a hallmark of aging is associated with marked increases in PAI-1 expression in tissues, is suggested as a bonafide marker and a critical mediator, of cellular senescence associated with aging and age-related diseases including cancer [22]. This study demonstrated that elevated level of PAI-1 is significantly associated with poor prognosis of cervical cancer, especially in the stage III patients treated without brachytherapy, further supporting the critical role of SASP in cervical cancer treatment outcome.

Production of pro-inflammatory mediators is a critical part of the SASP phenotype. Increased inflammation is widely known to play important roles in HPV-mediated cervical cancer [7,8,10,23]. Acute phase reactants (CRP and SAA) are pattern recognition molecules and are considered as part of innate immune system [24,25,26]. Both CRP and SAA are produced under the influence of the inflammatory cytokines, and can stimulate production of key SASP such as IL-8 [27], MMPs, chemokines (MCP-1), cytokines such as IL-6 and TNF-α, cytokine receptor antagonists [27,28,29,30].

The adipokine leptin is involved in energy homeostasis in healthy individuals, while in obesity leptin participates in the pro-inflammatory processes. In a meta-analysis of breast cancer study, higher leptin levels were associated with obesity and lymph node metastases [31]. Hyperactive leptin signaling has been implicated in pathogenesis and metastases in gynecological and breast cancers by inducing cell proliferation and reduces cell apoptosis by activating c-myc in cervical cancer [32,33]. Interestingly, high doses of leptin induce cell cycle arrest and senescence by activation of the p53/p21 pathway and inhibition of the SIRT1 pathway [34]. However, it has been reported that leptin can increase expression of PI3K/AKT/mTOR pathway and cell proliferation genes such as *cyclin D1*, *cyclin D2*, *cyclin D3* and *bcl-2*, and reduce the expression of p21, a senescence protein marker [35], suggesting a possible anti-senescence effect of leptin [36]. This is consistent with the role of lectin as a pivotal regulator for the control of food intake and energy expenditure, which are essential determinants of cellular senescence. In this study, higher leptin is marginally associated with better prognosis at individual protein level but contributes heavily to some prognostic models. Our observation is consistent with its proposed role as a senescence factor. Indeed, the role of leptin may depend on the concentration and route of administration, centrally or peripherally [37]. In supporting of this concept, moderate level of leptin is associated with better survival in our datasets. Given the variable roles and observations, the precise role of leptin in cancer remains to be resolved through additional clinical and experimental research.

The squamous cell carcinoma antigen (SCCA) is highly expressed in cervical cancer patients and other cancers such as hepatocellular carcinoma. We demonstrated previously in a large cohort that pretreatment SCCA is higher in late stage than early stage cervical cancer patients [38]. Several published studies have suggested that pretreatment serum SCCA is associated with recurrence [39,40,41] and normalization of SCCA after treatment is an indicator of good prognosis [41,42]. Here we presented strong evidence that stage II patients with higher pretreatment SCCA have worse prognosis and SCCA is a major contributor to the prognostic multi-protein models for RTBT2 patients. SCCA contains two isoforms in the serum, SCCA1 (SERPINB3) and SCCA2 (SERPINB4). They are members of the serine protease inhibitor (serpin) superfamily and SCCA1 may play a role in resistance to anti-cancer therapy [39,42]. SCCA may act as papain-like cysteine protease inhibitor to modulate host immune response against tumor cells and function as an inhibitor of UV-induced apoptosis. A recent study showed that SCCA1/2 are transcriptionally upregulated by oncogenic Ras and that increased SCCA expression leads to inhibition of protein turnover, unfolded protein response, activation of NF-kB and is essential for Ras-mediated cytokine production and tumor growth [43]. Analysis of human colorectal and pancreatic tumor samples reveals a positive correlation between Ras mutation, enhanced SCCA expression and IL-6 expression [43]. NF-kB is a key transcription factor for SASP and IL-6 is a major component of SASP [43]. These results indicate that SCCA is a Ras-responsive factor that is, at least partially, responsible for the observed cellular senescence phenotype in cervical cancer.

HGF is another important pro-senescence mediator by inducing p38 MAPK, AKT and NF-kB, which is a key senescence transcription factor. The receptor for HGF, cMET, is a well-known oncogene and a new senescence marker [44]. HGF is associated with an induction of mitochondrial oxidative stress, which in return contributes to HGF-dependent pro-senescence activity of ovarian cancer cells [45]. The senescence phenotype leads to oxidative stress, which in return promotes SASP, appearing to form an auto-regulatory loop.

While individual SASP proteins only have a modest impact on survival, our machine learning using Ridge regression discovered numerous multi-protein models that possess great potential to stratify patients into subsets with very different prognosis. Among the top 31 models discovered using patients in the RTBT2 dataset (stage III treated with EBRT+BT), all were validated by 1000 iterations of bootstrapping. All 31 models were also validated in the independent RTBT3 (stage 3 treated with EBRT+BT) dataset, despite the different stages between the two datasets, suggesting that these prognostic biomarkers are very robust and likely applicable to future samples.

This study also discovered 25 models using RT3 patients who are stage 3 and did not receive brachytherapy. All 25 top models were validated by 1000 iterations of bootstrapping and by the independent RTBT2 dataset. Furthermore, 22 of the 25 models were also validated in the independent RTBT3 dataset. These results suggest that these models are highly robust.

Together with our published analyses [11,12], it is abundantly clear that the prognosis of SCCC patients is determined primarily by at least three risk factors: stage, treatment modality and cellular senescence status. Stage 2 and stage 3 have a modest difference in survival (HR = 2.3). This report demonstrated that senescence is associated with poor survival in both stage 2 (HR = 3.09–4.52) and stage 3 (HR = 2.93–5.07) patients. Absence of brachytherapy was shown to be associated with a very poor survival (HR = 6.7) [11,12]; however, the analysis was likely confounded by the senescence status for patients who did and did not receive brachytherapy. After matching for senescence status, absence of brachytherapy is still associated with poor survival but only in SASP_H (HR = 3.3) and SASP_M (HR = 2.4) patient subsets. The best prognosis can be achieved using the combination of all three risk factors that seem to stratify all patients into four major survival categories. The best survival category is for Stage2-SASP_L-brachytherapy^+^ (BT^+^) (5yr survival ~80%); the next best survival category include is Stage2-SASP_H-BT^+^ and Stage3-SASP_L-BT^+^ patients (5yr survival ~55%); the next category includes Stage3-SASP_H-BT^+^ and Stage3-SASP_L-BT^−^ patients (5yr survival ~35%); and the worst survival category is Stage3-SASP_H-BT^−^ patients (5yr survival ~10%) (Figure 4d).

The interaction between brachytherapy and senescence is potentially of paramount importance clinically. We presented strong evidence that brachytherapy provides significant survival benefit to patients with moderate to high senescence. Therefore, patients with moderate to high senescence should be treated with brachytherapy to achieve the best outcome. It will be important to reassess whether brachytherapy should be given to patients who have low senescence because no significant benefit was seen for these patients. This could be an even more critical decision in resource-limited countries so that brachytherapy can be delivered to high senescence patients. Our results also raised an interesting possibility that the benefit of brachytherapy is primarily through killing senescent cells while external radiation therapy may not be efficient at eliminating senescent cells. This hypothesis is worth further investigation.

It has recently been shown that high intake of pro-inflammatory diet is associated with increased risk of cervical carcinogenesis [46]. Use of anti-inflammatory agents may improve the outcome of cancer chemotherapies such as carboplatin and gemcitabine [47]. Although anti-inflammatory therapies using pharmacological agents or nutritional supplements may be beneficial to cervical cancer treatment outcome, our data suggest that anti-inflammatory therapy may not be sufficient. The elevation in pro-inflammatory mediators is only a part of the cellular senescence phenotype, which is of critical importance is highlighted in this study. Therefore, reduction and elimination of senescent cells via pharmaceutical and/or nutritional senolytics such as the dasatinib and quercetin combination, which has been shown to eliminate senescent cells in a recent clinical trial [48], could be powerful strategies to further improve current chemoradiation therapies for cervical cancer and other cancers.

## 4. Materials and Methods

### 4.1. Study Design and Patients

This was a single-institution, prospective observational study examining serial serum samples in patients with cervical cancer. All the subjects included in this study were recruited from the Instituto Nacional de Enfermedades Neoplasicas, Lima, Peru, between 2004 and 2007. Informed consent was obtained from every subject or a legally authorized representative. Inclusion criteria were: (1) histologically confirmed squamous cell carcinoma, adeno-squamous carcinoma, or adenocarcinoma of the uterine cervix; (2) International Federation of Gynecology and Obstetrics (FIGO) stage II (≤4 cm), III, or IVA disease without rectal invasion; (3) measurable disease; (4) age between 20–75 years; (5) no prior surgery or chemotherapy for cervical cancer. Patients who had prior chemotherapy or pelvic radiotherapy were also excluded from the study. Venous blood was obtained from all subjects prior to initiation of treatment. Because the study was conducted between 2004–2007 it was not recorded if patients had stage IIIC disease. The patients then underwent treatment with pelvic external beam radiation (EBRT) alone or in combination with brachytherapy (EBRT + BT). The stage and grade of the tumors were determined according to the criteria established by the International Federation of Gynecology and Obstetrics. Disease-specific survival (DSS) was used as the clinical endpoints. The study was conducted according to the declaration of Helsinki (1996) and was approved by the institutional review boards at the Augusta University and the Instituto Nacional de Enfermedades Neoplasicas.

### 4.2. Processing of Blood Samples

Venous blood collected in serum separator tubes (BD Biosciences, San Jose, CA, USA) was allowed to clot for 30 min at room temperature. Serum was separated by centrifuging at 2000× *g* at 20 °C. Aliquots of serum were prepared immediately after into wells of 96-well plates (150 µL/well) to create master plates. Daughter plates were then created by pipetting 5–25 µL of serum/well to avoid repeated freeze/thaw for all samples. Samples were aliquoted and stored in a –80 °C freezer until use. For each measurement, one daughter plate was thawed and used for the serum measurement.

### 4.3. Luminex Multiplex Protein Assay

We selected 19 proteins, viz serum amyloid A (SAA), C-reactive protein (CRP), CXCL chemokines (MIG and GRO-α), soluble cytokine receptors (sIL1RII, sIL2Rα, sIL6R, sTNFRI and sTNFRII), growth factors derived from epithelial (sEGFR), hepatocyte (HGF) and platelets (PDGF.AA and PDGF.ABBB), squamous cell carcinoma antigen (SCCA), insulin-like growth factor binding protein 2 (IGFBP 2), tissue Plasminogen activator inhibitor-1 (tPAI1), matrix-metalloproteinase 1 (MMP1), leptin, adhesion molecule (sE-selectin), to be measured in the serum [38]. These proteins were examined for their ability to predict DSS and PFS when drawn at the time of diagnosis.

Luminex assays for the above mentioned 19 proteins were obtained from Millipore Inc. (Billerica, MA, USA). Multiplex assays were performed according to instructions provided with the kit. Serum samples were incubated with capture antibodies immobilized on polystyrene beads for one hour. The beads were then washed and further incubated with biotinylated detection antibody cocktail for one hour. Next, beads were washed twice to remove unbound detection antibody, and then incubated with phycoerythrin-labeled streptavidin for thirty minutes. Last, beads were washed and suspended in 60 µL of wash buffer.

The median fluorescence intensities (MFI) were measured using a FlexMAP 3D array reader (Millipore) with the following instrument settings: events/bead: 50, minimum events: 0, flow rate: 60 µL/min, Sample size: 50 μL and discriminator gate: 8000–13,500.

Luminex median fluorescence intensity (MFI) data was subjected to quality control analysis for low bead counts, high bead CV [49]. The coefficient of variation of replicate wells was also checked and wells with CV > 25% were not included in further analyses.

Protein concentrations for samples were estimated using a regression fit to the standard curve with known concentration included on each plate using a serial dilution series. To achieve normal distribution, MFI and concentrations for standards were log2 transformed prior to all statistical analyses.

### 4.4. Statistical Analysis

All statistical analyses were performed using the R language and environment for statistical computing (R version 3.62; R Foundation for Statistical Computing; www.r-project.org, accessed on 20 December 2019). The protein concentrations were log2 normalized after initial QC. The statistical significance of differences was set at *p* < 0.05, all *p* values were two sided. Patients with no history of recurrence or death were censored at the date of last follow-up visit. Patients who died of natural causes unrelated to cancer were censored at time of death. DSS and PFS for all subjects greater than 5 years was censored at 5 years. Kaplan-Meier survival analysis and log-rank test were used to compare differences in DSS between patients in different quartiles using the 1st quartile as reference. Cox proportional hazards analyses were used to assess survival. Effect of co-variates such as stage, treatment type and protein level on disease specific (DSS) was evaluated by adding in Cox proportional hazards models.

In order to create a comprehensive multivariate score that serum data, we used the elastic net algorithm (R package glmnet) [50]. This algorithm combines multiple predictors in a linear combination and tunes the model base on a penalty term, which is the sum of the square of the coefficients used in the model. The effect of the penalty term can be adjusted to either have no effect lambda = 0 or as lambda approaches infinity, variable coefficients approach 0. The sum of the linear combination yields a composite score for each individual patient. The number of predictors is optimized by setting the alpha value to 0, where an alpha = 0 includes all variables added to the glmnet model. The optimum lambda was determined using the lambda.min function in R, which automatically chooses the best lambda value to eliminate errors on cross validation. The composite score of the combined predictors for each value of alpha were then subjected to survival analysis and cox proportional hazards to determine the best score for predicting DSS.

## 5. Conclusions

The interaction between brachytherapy and senescence is potentially of paramount importance clinically. We presented strong evidence that brachytherapy provides significant survival benefit to patients with moderate to high senescence. Therefore, patients with moderate to high senescence should be treated with brachytherapy to achieve the best outcome. It will be important to reassess whether brachytherapy should be given to patients who have low senescence because no significant benefit was seen for these patients. This could be an even more critical decision in resource-limited countries so that brachytherapy can be delivered to high senescence patients. Our results also raised an interesting possibility that the benefit of brachytherapy is primarily through killing senescent cells while external radiation therapy may not be efficient at eliminating senescent cells. This hypothesis is worth of further investigation.

## Figures and Tables

**Table 1:** Clinical and demographic variables for subjects with squamous cell carcinoma of cervix (*n* = 565) recruited in Cervicusco study.

Variable	Stage IIB (*n* = 276)	Stage IIIB (*n* = 289)	*p*-Value
Age at Diagnosis (Years)	48.67	51.14	0.0025
Range (Years)	26–76	27–82	
<50 Years (*n*)	171	140	
>50 Years (*n*)	105	149	
Median age (Years)	48	51	0.0015 ^1^
Median DSS (Years)			
EBRT	NA	0.99	2.3 × 10^−26^
EBRT +BT	NYR ^2^	4.8	
Treatment type (*n*)			
EBRT		86	2.3 × 10^−22^
EBRT +BT	276	203	
Reccurence Sites			
No information	223	241	
Other Organs	25	12	
Pelvis	12	21	

^1^ Kruskall-Wallis test, ^2^ NYR: not yet reached, EBRT: External beam radiotherapy, BT: Brachytherapy, significance for differences in median DSS was computed by chi-square test.

**Table 2:** Cox proportional hazards ratio (95% CI) for individual proteins after adjusting for stage and treatment as co-variates.

Protein	Quartile (*n*)	HR_Q2	*p*_ Q2	HR_Q3	*p*_ Q3	HR_Q4	*p*_ Q4
CRP	139/149/144/146	1.16 (0.77–1.76)	0.473	1.2 (0.78–1.84)	0.4	1.89 (1.27–2.81)	0.00162
GRO	139/140/131/168	0.879 (0.55–1.40)	0.585	1.52 (1.00–2.32)	0.0517	2.07 (1.40–3.07)	0.000273
HGF	132/148/146/152	1.29 (0.85–1.96)	0.23	0.979 (0.63–1.52)	0.925	1.78 (1.18–2.69)	0.00631
IGFBP2	145/144/144/145	1.12 (0.74–1.68)	0.594	1.19 (0.81–1.77)	0.378	1.22 (0.82–1.83)	0.327
LEPTIN	145/144/144/145	0.78 (0.53–1.14)	0.194	0.70 (0.48–1.02)	0.0601	0.61 (0.41–0.90)	0.014
MIG	146/144/144/144	1.34 (0.89–2.02)	0.16	1.32 (0.87–2.02)	0.193	1.85 (1.23–2.77)	0.00284
MMP1	146/144/144/144	0.891 (0.58–1.36)	0.593	1.36 (0.92–2.01)	0.122	1.69 (1.15–2.48)	0.00778
PDGFAA	145/144/144/145	1.02 (0.69–1.52)	0.914	0.86 (0.57–1.29)	0.47	1.35 (0.92–1.98)	0.127
PDGFAA/AB	145/144/144/145	0.77 (0.52–1.15)	0.199	1.03 (0.71–1.50)	0.881	1.03 (0.70–1.52)	0.876
SAA	146/144/144/144	0.889 (0.59–1.34)	0.573	1.12 (0.75–1.68)	0.577	1.59 (1.08–2.36)	0.02
SCCA	145/144/144/145	1.26 (0.82–1.93)	0.292	1.36 (0.89–2.07)	0.156	1.38 (0.90–2.11)	0.139
sE.Selectin	146/144/144/144	0.757 (0.51–1.13)	0.171	0.974 (0.66–1.43)	0.891	1.09 (0.75–1.58)	0.642
sEGFR	145/144/144/145	0.727 (0.50–1.06)	0.101	0.93 (0.64–1.36)	0.707	0.696 (0.48–1.02)	0.0602
sIL1RII	145/144/144/145	0.774 (0.53–1.13)	0.183	0.734 (0.50–1.07)	0.105	0.737 (0.50–1.08)	0.118
sIL2Rα	145/144/144/145	0.947 (0.62–1.44)	0.8	1.32 (0.89–1.96)	0.169	1.43 (0.96–2.13)	0.0795
sIL6R	146/144/144/144	1.1 (0.75–1.60)	0.624	1 (0.68–1.47)	0.985	0.822 (0.55–1.23)	0.336
sTNFRI	146/144/144/144	0.913 (0.61–1.37)	0.66	1.25 (0.85–1.83)	0.258	1.04 (0.70–1.55)	0.84
sTNFRII	145/144/144/145	0.717 (0.47–1.08)	0.113	0.87 (0.59–1.28)	0.479	1.15 (0.78–1.69)	0.489
tPAI1	145/144/144/145	1.23 (0.82–1.86)	0.316	1.07 (0.71–1.62)	0.74	1.79 (1.22–2.62)	0.00269

Protein levels were divided into 4 quartiles containing 25% subjects. Quartile 1 was compared to quartile 2–4. *p* < 0.05 was considered significant. *n*: number of individuals in each quartile.

**Table 3:** Cox proportional hazards ratio (95% CI) for 10 indvidual proteins for three subgroups based on stage and treatment type.

Model Number	RTBT2 Training & Testing				RTBT2 Bootstrapping (1000)				RTBT3 Data Set	
	Train HR	Train_*p*	HR_Test	Test_ *p*	Mean HR	*p* > 0.05	*p* = 0.05–0.001	*p* < 0.001	HR (95% CI)	*p* Value
**RTBT2: 8 Protein models**										
1395	3.07 (1.34–7.05)	0.00826	5.66 (1.73–18.51)	0.00417	4.05 (2.63–7.07)	1	496	503	2 (1.27–3.15)	0.00264
525	3.19 (1.47–6.92)	0.0034	3.26 (1.32–8.05)	0.0103	3.37 (2.31–5.24)	1	498	501	2.21 (1.42–3.45)	0.000478
1670	3.15 (1.37–7.28)	0.00703	3.04 (1.38–6.7)	0.00572	3.17 (2.22–4.74)	1	566	433	2.32 (1.49–3.61)	0.00018
33	3.21 (1.40–7.34)	0.00588	4.14 (1.45–11.77)	0.00777	3.71 (2.49–6.02)	2	583	415	2.02 (1.29–3.19)	0.00231
1390	3.06 (1.41–6.66)	0.00481	3.23 (1.31–7.93)	0.0106	3.25 (2.24–4.8)	1	593	406	2.33 (1.49–3.64)	0.000196
406	3.02 (1.31–6.94)	0.00923	3.30 (1.43–7.58)	0.00494	3.24 (2.26–4.81)	2	596	402	2.08 (1.34–3.24)	0.00109
1055	3.21 (1.40–7.35)	0.00584	3.56 (1.38–9.24)	0.00889	3.49 (2.35–5.45)	2	606	392	2.12 (1.35–3.32)	0.00103
152	3.27 (1.34–8.01)	0.00947	3.01 (1.32–6.83)	0.00854	3.27 (2.23–5.02)	1	661	338	2.52 (1.63–3.9)	3.59 × 10^−5^
1472	3.63 (1.38–9.55)	0.00902	3.22 (1.35–7.66)	0.00839	3.61 (2.44–5.76)	1	661	338	2.45 (1.58–3.8)	5.84 × 10^−5^
1241	3.11 (1.26–7.64)	0.0134	3.00 (1.38–6.53)	0.00559	3.15 (2.22–4.73)	2	675	323	2.08 (1.33–3.25)	0.00142
679	3.03 (1.38–6.64)	0.00559	3.13 (1.28–7.62)	0.0122	3.13 (2.19–4.84)	3	703	294	2.34 (1.51–3.63)	0.000139
636	3.21 (1.41–7.34)	0.00558	3.02 (1.24–7.34)	0.0148	3.17 (2.12–4.74)	7	726	267	2.32 (1.48–3.61)	0.000218
1574	3.98 (1.64–9.65)	0.00225	4.23 (1.49–11.98)	0.00668	4.36 (2.8–7.32)	6	994	8	2.04 (1.3–3.2)	0.00186
142	5.11 (1.98–13.19)	0.000741	3.09 (1.19–8.0)	0.0203	4.20 (2.82–6.88)	7	993	8	2.19 (1.4–3.42)	0.000553
804	4.22 (1.62–10.99)	0.00324	4.13 (1.46–11.66)	0.00742	4.52 (2.91–7.85)	7	993	8	2.33 (1.49–3.66)	0.000226
54	4.13 (1.72–9.92)	0.00154	3.24 (1.34–7.89)	0.00938	3.82 (2.56–5.96)	11	989	8	2.13 (1.36–3.34)	0.000962
599	4.64 (1.8–11.96)	0.00149	3.05 (1.26–7.4)	0.0135	3.90 (2.58–5.96)	15	985	8	2.1 (1.34–3.28)	0.00122
**RTBT2: 7 Protein models**										
894	3.39 (1.39–8.24)	0.00707	3.63 (1.59–8.29)	0.00222	3.67 (2.52–5.74)	1	356	643	2.55 (1.64–3.97)	3.23 × 10^−5^
242	3.73 (1.55–8.99)	0.00334	3.03 (1.37–6.72)	0.00638	3.49 (2.4–5.29)	2	403	595	2.3 (1.48–3.6)	0.000245
206	3.31 (1.45–7.57)	0.00447	3.55 (1.46–8.63)	0.00515	3.56 (2.41–5.65)	1	447	552	2.38 (1.52–3.72)	0.000138
1713	3.32 (1.36–8.07)	0.00814	3.02 (1.37–6.62)	0.00593	3.22 (2.24–4.74)	1	611	388	2.14 (1.37–3.34)	0.000791
871	3.11 (1.43–6.75)	0.00416	6.72 (1.59–28.33)	0.00943	3.85 (2.52–6.35)	2	636	362	2.15 (1.36–3.38)	0.000975
29	3.83 (1.68–8.72)	0.00136	3.18 (1.11–9.12)	0.0312	3.51 (2.28–5.66)	2	734	264	2.06 (1.32–3.24)	0.00155
1875	3.26 (1.42–7.49)	0.00548	3.64 (1.28–10.32)	0.0153	3.46 (2.29–5.46)	4	742	254	2.08 (1.32–3.3)	0.00174
897	3.02 (1.32–6.91)	0.00901	3.12 (1.29–7.55)	0.0118	3.14 (2.16–4.89)	4	754	242	2.33 (1.48–3.65)	0.000239
841	3.20 (1.4–7.31)	0.00571	3.30 (1.27–8.59)	0.0142	3.29 (2.21–5.23)	4	763	233	2.09 (1.33–3.27)	0.00129
599	3.00 (1.31–6.87)	0.00929	3.03 (1.25–7.34)	0.0141	3.09 (2.11–4.69)	2	805	193	2.09 (1.33–3.27)	0.00129
859	3.03 (1.32–6.94)	0.0088	3.23 (1.25–8.36)	0.0158	3.23 (2.13–5.33)	6	803	191	2.41 (1.54–3.76)	0.000111
1229	3.24 (1.33–7.87)	0.0095	3.67 (1.3–10.39)	0.0142	3.56 (2.33–5.85)	6	804	190	2.73 (1.76–4.23)	7.27 × 10^−6^
15	3.36 (1.38–8.18)	0.00743	4.64 (1.8–11.94)	0.00147	4.20 (2.83–6.8)	4	996	0	2.21 (1.4–3.48)	0.000615
1579	4.04 (1.68–9.72)	0.0018	3.64 (1.57–8.43)	0.00251	3.96 (2.66–6.25)	6	994	0	2.23 (1.43–3.48)	0.000414

Protein levels were divided into 4 quartiles containing 25% subjects. Quartile 1 was compared to quartile 2–4. *p* < 0.05 was considered significant, n: number of individuals in each quartile.

**Table 4:** Summary of bootstrapping results showing HR (95%) and number of models having *p* values < 0.05 for selected models for 7 protein models and 8 protein models for Group 1–3.

Model Number	RT3 Training & Testing				RT3 Bootstrapping (1000)				RTBT2 Data Set		RTBT3 Data Set	
	Train HR	Train_*p*	HR_Test	Test_ *p*	Mean HR	*p* > 0.05	*p* = 0.05–0.001	*p* < 0.001	HR (95% CI)	*p* Value	HR (95% CI)	*p* Value
**RT3: 8 Protein models**									g			
54	3.92 (1.65–9.3)	0.00199	3.46 (1.37–8.72)	0.00868	3.65 (2.45–6.17)	1	371	628	1.92 (1.01–3.66)	0.0482	2.48 (1.6–3.86)	5.48 × 10^−5^
179	3.54 (1.44–8.73)	0.00602	3.16 (1.32–7.54)	0.00961	3.39 (2.25–5.33)	4	591	405	2 (1.2–3.33)	0.00749	2.2 (1.4–3.45)	0.000582
727	3.76 (1.38–10.22)	0.0094	3.19 (1.33–7.65)	0.00917	3.61 (2.39–5.5)	4	624	372	1.94 (1.2–3.14)	0.00678	1.54 (0.98–2.43)	0.0596
85	3.85 (1.56–9.48)	0.00338	3.81 (1.28–11.31)	0.016	3.87 (2.53–5.96)	4	635	361	2.01 (1.23–3.29)	0.00508	1.85 (1.17–2.93)	0.00852
1590	3.18 (1.23–8.21)	0.017	3.56 (1.54–8.27)	0.00308	3.4 (2.27–5.21)	4	659	337	2.08 (1.22–3.57)	0.00758	2.59 (1.67–4.01)	2.08 × 10^−5^
126	3.27 (1.35–7.91)	0.00868	4.03 (1.5–10.82)	0.00572	3.44 (2.22–5.42)	10	707	283	2.02 (1.24–3.28)	0.00463	2.11 (1.34–3.33)	0.00126
345	3.4 (1.39–8.32)	0.00736	3.04 (1.26–7.36)	0.0134	3.04 (2–4.91)	18	759	223	1.91 (1.11–3.27)	0.019	2.19 (1.4–3.41)	0.000551
1058	3.19 (1.32–7.68)	0.00979	3.25 (1.26–8.4)	0.0147	3.26 (2.23–4.9)	7	778	215	2.4 (1.46–3.96)	0.00055	2 (1.28–3.12)	0.00224
1205	3.81 (1.62–8.94)	0.00216	3.71 (1.34–10.23)	0.0115	3.1 (2.06–4.84)	19	784	197	2.1 (1.3–3.37)	0.00231	1.64 (1.05–2.57)	0.0298
1539	5.42 (2.08–14.13)	0.00054	3.22 (1.07–9.66)	0.0374	3.12 (2.06–4.8)	24	812	164	2.5 (1.55–4.02)	0.00016	1.52 (0.96–2.4)	0.0725
1430	3.71 (1.47–9.34)	0.00543	3.35 (1.12–10.04)	0.031	3.22 (2.14–5.09)	23	862	115	1.99 (1.22–3.23)	0.00565	1.23 (0.77–1.96)	0.396
1908	3.28 (1.37–7.86)	0.00763	3.11 (1.15–8.45)	0.0259	3.05 (1.96–4.72)	43	856	101	2.36 (1.44–3.87)	0.00062	1.82 (1.17–2.85)	0.00794
435	3.27 (1.44–7.47)	0.00482	3.06 (1.02–9.2)	0.0468	3.03 (2.01–4.68)	41	865	94	2.34 (1.44–3.81)	0.00059	1.66 (1.06–2.61)	0.028
384	5.93 (2.48–14.17)	6.25E-05	4.19 (1.39–12.64)	0.011	4.61 (3–7.28)	2	998	8	1.99 (1.24–3.2)	0.00455	2.07 (1.33–3.22)	0.00119
1464	4.24 (1.57–11.46)	0.00443	3.89 (1.43–10.56)	0.00767	4.52 (2.92–7.61)	14	986	8	1.92 (1.19–3.12)	0.00803	2 (1.28–3.11)	0.0023
304	4.28 (1.64–11.16)	0.00292	5.46 (2.21–13.49)	0.00023	3.78 (2.54–6.08)	19	981	8	1.96 (1.09–3.53)	0.025	2.63 (1.7–4.08)	1.48 × 10^−5^
1722	3.42 (1.47–7.99)	0.00443	3.56 (1.37–9.27)	0.00941	3.58 (2.4–5.84)	38	962	8	2.01 (1.13–3.57)	0.0172	2.53 (1.63–3.93)	3.80 × 10^−5^
**RT3: 7 Protein models**												
1689	7.54 (2.69–21.11)	0.00012	3.91 (1.51–10.13)	0.00493	5.07 (3.32–8.27)	0	45	955	2.03 (1.24–3.33)	0.0048	2.29 (1.47–3.58)	0.00026
775	5.51 (2.13–14.23)	0.00042	3.54 (1.51–8.26)	0.0035	4.61 (3.09–7.19)	0	67	933	2.44 (1.46–4.09)	0.0006	2.09 (1.33–3.29)	0.00149
10	3.88 (1.59–9.47)	0.00287	3.49 (1.36–8.95)	0.00928	3.9 (2.74–5.91)	0	249	751	1.91 (1.08–3.39)	0.027	2.22 (1.42–3.48)	0.000499
1969	3.95 (1.64–9.49)	0.00215	5.23 (1.51–18.16)	0.00919	4.34 (2.79–6.77)	0	392	608	1.98 (1.22–3.21)	0.0056	2.43 (1.56–3.78)	8.30 × 10^−5^
1263	3.41 (1.44–8.05)	0.00512	3.65 (1.4–9.51)	0.00815	3.61 (2.35–5.55)	1	559	440	2.04 (1.19–3.49)	0.0097	2.17 (1.39–3.4)	0.000713
345	4.45 (1.73–11.48)	0.002	3.07 (1.27–7.42)	0.0126	3.41 (2.3–5.16)	1	569	430	2.42 (1.46–4.01)	0.00058	2.34 (1.51–3.64)	0.000158
304	4.28 (1.64–11.16)	0.00292	3.34 (1.4–7.94)	0.00646	3.5 (2.33–5.64)	4	612	384	2 (1.13–3.55)	0.018	2.52 (1.62–3.92)	4.02 × 10^−5^
1644	3.05 (1.21–7.68)	0.0181	3.24 (1.36–7.7)	0.00777	2.93 (1.97–4.58)	32	768	200	2.05 (1.16–3.64)	0.0141	2.37 (1.52–3.68)	0.000129

Number of models counted for *p* value intervals in the table.

**Table 5:** Impact of SASP status on response to brachytherapy.

Model Number	SASP_L				SASP_H			
	# Patients (+BT/−BT)	HR (95% CI)	*p* Value	Adj.p	# Patients (+BT/−BT)	HR (95% CI)	*p* Value	Adj.p
#7-10	151/43	1.95 (1.15–3.32)	0.014	0.34	52/43	3.06 (1.78–5.26)	5.27 × 10^−5^	0.00131723
#7-1263	152/35	1.79 (0.99–3.21)	0.052	1.30	51/51	2.47 (1.48–4.14)	0.00057642	0.0144105
#7-1644	153/38	2.25 (1.34–3.79)	0.002	0.06	50/48	2.34 (1.37–3.98)	0.00177445	0.04436126
#7-1689	141/40	1.58 (0.88–2.86)	0.126	3.15	62/46	3.70 (2.22–6.17)	5.28 × 10^−7^	1.32 × 10^−5^
#7-1969	131/29	1.56 (0.81–3.00)	0.184	4.61	72/57	2.99 (1.87–4.79)	5.04 × 10^−6^	0.00012591
#7-304	146/34	2.01 (1.11–3.64)	0.020	0.51	57/52	2.27 (1.39–3.70)	0.001019163	0.02547908
#7-345	144/39	1.99 (1.14–3.49)	0.016	0.41	59/47	2.85 (1.72–4.71)	4.43 × 10^−5^	0.0011083
#7-775	152/39	1.74 (1.00–3.02)	0.051	1.28	51/47	3.24 (1.89–5.54)	1.89 × 10^−5^	0.00047331
#8-1058	135/34	1.85 (1.02–3.35)	0.043	1.06	68/52	3.09 (1.90–5.03)	5.81 × 10^−6^	0.00014527
#8-1205	114/33	2.22 (1.22–4.03)	0.009	0.23	89/53	3.16 (2.00–5.01)	9.57 × 10^−7^	2.39 × 10^−5^
#8-126	139/36	1.77 (0.98–3.20)	0.057	1.43	64/50	3.10 (1.88–5.11)	8.81 × 10^−6^	0.00022014
#8-1430	101/28	1.76 (0.92–3.39)	0.090	2.25	102/58	3.61 (2.31–5.66)	1.92 × 10^−8^	4.80 × 10^−7^
#8-1464	101/31	1.41 (0.70–2.84)	0.338	8.45	102/55	4.07 (2.61–6.32)	4.82 × 10^−10^	1.20 × 10^−8^
#8-1539	114/32	2.01 (1.09–3.68)	0.025	0.63	89/54	3.22 (2.04–5.11)	6.10 × 10^−7^	1.53 × 10^−5^
#8-1590	144/34	1.96 (1.08–3.55)	0.026	0.66	59/52	2.56 (1.58–4.16)	0.000145401	0.00363503
#8-1722	158/40	2.10 (1.25–3.53)	0.005	0.12	45/46	2.37 (1.37–4.10)	0.002131359	0.05328398
#8-179	149/40	2.00 (1.17–3.40)	0.011	0.27	54/46	2.98 (1.75–5.09)	6.10 × 10^−5^	0.00152563
#8-1908	103/31	1.70 (0.93–3.12)	0.085	2.13	100/55	4.15 (2.58–6.66)	3.83 × 10^−9^	9.58 × 10^−8^
#8-304	153/39	2.05 (1.19–3.53)	0.010	0.25	50/47	2.43 (1.45–4.10)	0.000817003	0.02042507
#8-345	144/38	2.19 (1.26–3.79)	0.005	0.13	59/48	2.56 (1.55–4.24)	0.00025089	0.00627225
#8-384	118/36	1.68 (0.90–3.12)	0.103	2.58	85/50	3.89 (2.43–6.24)	1.61 × 10^−8^	4.04 × 10^−7^
#8-435	115/31	1.79 (0.98–3.26)	0.058	1.46	88/55	3.61 (2.24–5.83)	1.49 × 10^−7^	3.73 × 10^−6^
#8-54	164/43	2.21 (1.36–3.60)	0.001	0.04	39/43	2.52 (1.40–4.54)	0.001995723	0.04989307
#8-727	112/35	1.69 (0.92–3.09)	0.089	2.21	91/51	4.14 (2.56–6.68)	6.04 × 10^−9^	1.51 × 10^−7^
#8-85	132/29	1.67 (0.89–3.14)	0.112	2.80	71/57	2.95 (1.82–4.77)	1.03 × 10^−5^	0.00025838

Adj.p: adjusted *p*-value, +BT: radiotherapy+brachytherapy, −BT: radiotherapy alone.

## References

[1] Bray F., Ferlay J., Soerjomataram I., Siegel R.L., Torre L.A., Jemal A. (2018). Global cancer statistics 2018: GLOBOCAN estimates of incidence and mortality worldwide for 36 cancers in 185 countries. CA Cancer J. Clin..

[2] Walboomers J.M., Jacobs M.V., Manos M.M., Bosch F.X., Kummer J.A., Shah K.V., Snijders P.J., Peto J., Meijer C.J., Munoz N. (1999). Human papillomavirus is a necessary cause of invasive cervical cancer worldwide. J. Pathol..

[3] Stanley M.A., Pett M.R., Coleman N. (2007). HPV: From infection to cancer. Biochem. Soc. Trans..

[4] Koromilas A.E., Li S., Matlashewski G. (2001). Control of interferon signaling in human papillomavirus infection. Cytokine Growth Factor Rev..

[5] Sales K.J., Katz A.A. (2012). Inflammatory pathways in cervical cancer—The UCT contribution. S. Afr. Med. J..

[6] Łaniewski P., Cui H., Roe D.J., Barnes D., Goulder A., Monk B.J., Greenspan D.L., Chase D.M., Herbst-Kralovetz M.M. (2019). Features of the cervicovaginal microenvironment drive cancer biomarker signatures in patients across cervical carcinogenesis. Sci. Rep..

[7] Hemmat N., Bannazadeh Baghi H. (2019). Association of human papillomavirus infection and inflammation in cervical cancer. Pathog. Dis..

[8] Castle P.E., Hillier S.L., Rabe L.K., Hildesheim A., Herrero R., Bratti M.C., Sherman M.E., Burk R.D., Rodriguez A.C., Alfaro M. (2001). An association of cervical inflammation with high-grade cervical neoplasia in women infected with oncogenic human papillomavirus (HPV). Cancer Epidemiol. Biomark. Prev..

[9] Chen H.H., Su W.C., Chou C.Y., Guo H.R., Ho S.Y., Que J., Lee W.Y. (2005). Increased expression of nitric oxide synthase and cyclooxygenase-2 is associated with poor survival in cervical cancer treated with radiotherapy. Int. J. Radiat. Oncol. Biol. Phys..

[10] Fernandes J.V., De Medeiros Fernandes T.A.A., De Azevedo J.C.V., Cobucci R.N.O., De Carvalho M.G.F., Andrade V.S., De Araújo J.M.G. (2015). Link between chronic inflammation and human papillomavirus-induced carcinogenesis (Review). Oncol. Lett..

[11] Purohit S., Ferris D.G., Alvarez M., Tran P.M.H., Tran L.K.H., Mysona D.P., Hopkins D., Zhi W., Dun B., Wallbillich J.J. (2020). Better survival is observed in cervical cancer patients positive for specific anti-glycan antibodies and receiving brachytherapy. Gynecol. Oncol..

[12] Tran L.K.H., Tran P.M.H., Mysona D.P., Purohit S.B., Myers E., Lee W.S., Dun B., Xu D., Liu H., Hopkins D. (2020). A 73-gene proliferative transcriptomic signature predicts uterine serous carcinoma patient survival and response to primary therapy. Gynecol. Oncol..

[13] Coppé J.P., Desprez P.Y., Krtolica A., Campisi J. (2010). The senescence-associated secretory phenotype: The dark side of tumor suppression. Annu. Rev. Pathol..

[14] Stone S.C., Rossetti R.A.M., Lima A.M., Lepique A.P. (2014). HPV associated tumor cells control tumor microenvironment and leukocytosis in experimental models. Immun. Inflamm. Dis..

[15] Chow M.T., Luster A.D. (2014). Chemokines in Cancer. Cancer Immunol. Res..

[16] Perillo N.L., Naeim F., Walford R.L., Effros R.B. (1993). The in vitro senescence of human T lymphocytes: Failure to divide is not associated with a loss of cytolytic activity or memory T cell phenotype. Mech. Ageing Dev..

[17] Hildesheim A., Schiffman M.H., Tsukui T., Swanson C.A., Lucci J., Scott D.R., Glass A.G., Rush B.B., Lorincz A.T., Corrigan A. (1997). Immune activation in cervical neoplasia: Cross-sectional association between plasma soluble interleukin 2 receptor levels and disease. Cancer Epidemiol. Biomark. Prev..

[18] Ung A., Kramer T.R., Schiffman M., Herrero R., Bratti M.C., Burk R.D., Swanson C.A., Sherman M.E., Hutchinson M.L., Alfaro M. (1999). Soluble interleukin 2 receptor levels and cervical neoplasia: Results from a population-based case-control study in Costa Rica. Cancer Epidemiol. Biomark. Prev..

[19] Tsukui T., Hildesheim A., Schiffman M.H., Lucci J., Contois D., Lawler P., Rush B.B., Lorincz A.T., Corrigan A., Burk R.D. (1996). Interleukin 2 production in vitro by peripheral lymphocytes in response to human papillomavirus-derived peptides: Correlation with cervical pathology. Cancer Res..

[20] Sheu B.C., Hsu S.M., Ho H.N., Lien H.C., Huang S.C., Lin R.H. (2001). A novel role of metalloproteinase in cancer-mediated immunosuppression. Cancer Res..

[21] Kobayashi A., Weinberg V., Darragh T., Smith-McCune K. (2008). Evolving immunosuppressive microenvironment during human cervical carcinogenesis. Mucosal Immunol..

[22] Vaughan D.E., Rai R., Khan S.S., Eren M., Ghosh A.K. (2017). Plasminogen Activator Inhibitor-1 Is a Marker and a Mediator of Senescence. Arterioscler. Thromb. Vasc. Biol..

[23] Ames B.N., Gold L.S., Willett W.C. (1995). The causes and prevention of cancer. Proc. Natl. Acad. Sci. USA.

[24] Marnell L., Mold C., Du Clos T.W. (2005). C-reactive protein: Ligands, receptors and role in inflammation. Clin. Immunol..

[25] Black S., Kushner I., Samols D. (2004). C-reactive Protein. J. Biol. Chem..

[26] Volanakis J.E. (2001). Human C-reactive protein: Expression, structure, and function. Mol. Immunol..

[27] Furlaneto C.J., Campa A. (2000). A novel function of serum amyloid A: A potent stimulus for the release of tumor necrosis factor-alpha, interleukin-1beta, and interleukin-8 by human blood neutrophil. Biochem. Biophys. Res. Commun..

[28] Rhodes B., Fürnrohr B.G., Vyse T.J. (2011). C-reactive protein in rheumatology: Biology and genetics. Nat. Rev. Rheumatol..

[29] Malle E., De Beer F.C. (1996). Human serum amyloid A (SAA) protein: A prominent acute-phase reactant for clinical practice. Eur. J. Clin. Investig..

[30] Sproston N.R., Ashworth J.J. (2018). Role of C-Reactive Protein at Sites of Inflammation and Infection. Front. Immunol..

[31] Gu L., Wang C.-D., Cao C., Cai L.-R., Li D.-H., Zheng Y.-Z. (2019). Association of serum leptin with breast cancer: A meta-analysis. Medicine.

[32] Yuan Y., Zhang J., Cai L., Ding C., Wang X., Chen H., Wang X., Yan J., Lu J. (2013). Leptin induces cell proliferation and reduces cell apoptosis by activating c-myc in cervical cancer. Oncol. Rep..

[33] Crean-Tate K.K., Reizes O. (2018). Leptin Regulation of Cancer Stem Cells in Breast and Gynecologic Cancer. Endocrinology.

[34] Zhao X., Dong Y., Zhang J., Li D., Hu G., Yao J., Li Y., Huang P., Zhang M., Zhang J. (2016). Leptin changes differentiation fate and induces senescence in chondrogenic progenitor cells. Cell Death Dis..

[35] Wen R., Hu S., Xiao Q., Han C., Gan C., Gou H., Liu H., Li L., Xu H., He H. (2015). Leptin exerts proliferative and anti-apoptotic effects on goose granulosa cells through the PI3K/Akt/mTOR signaling pathway. J. Steroid Biochem. Mol. Biol..

[36] Zhan K., Ning M., Wang C., Tang Y., Gu H., Yan C., Tang X. (2016). Formaldehyde accelerates cellular senescence in HT22 cells: Possible involvement of the leptin pathway. Acta Biochim. Biophys. Sin..

[37] Sharma A., Bartell S.M., Baile C.A., Chen B., Podolsky R.H., McIndoe R.A., She J.X. (2010). Hepatic gene expression profiling reveals key pathways involved in leptin-mediated weight loss in ob/ob mice. PLoS ONE.

[38] Zhi W., Ferris D., Sharma A., Purohit S., Santos C., He M., Ghamande S., She J.X. (2014). Twelve serum proteins progressively increase with disease stage in squamous cell cervical cancer patients. Int. J. Gynecol. Cancer.

[39] Rose P.G., Baker S., Fournier L., Nelson B.E., Hunter R.E. (1993). Serum squamous cell carcinoma antigen levels in invasive cervical cancer: Prediction of response and recurrence. Am. J. Obstet. Gynecol..

[40] Ohara K., Tanaka Y., Tsunoda H., Nishida M., Sugahara S., Itai Y. (2002). Assessment of cervical cancer radioresponse by serum squamous cell carcinoma antigen and magnetic resonance imaging. Obstet. Gynecol..

[41] Markovina S., Wang S., Henke L.E., Luke C.J., Pak S.C., DeWees T., Pfeifer J.D., Schwarz J.K., Liu W., Chen S. (2018). Serum squamous cell carcinoma antigen as an early indicator of response during therapy of cervical cancer. Br. J. Cancer.

[42] Chen P., Jiao L., Wang D.B. (2017). Squamous cell carcinoma antigen expression in tumor cells is associated with the chemosensitivity and survival of patients with cervical cancer receiving docetaxel-carboplatin-based neoadjuvant chemotherapy. Oncol. Lett..

[43] Catanzaro J.M., Sheshadri N., Pan J.A., Sun Y., Shi C., Li J., Powers R.S., Crawford H.C., Zong W.X. (2014). Oncogenic Ras induces inflammatory cytokine production by upregulating the squamous cell carcinoma antigens SerpinB3/B4. Nat. Commun..

[44] Boichuck M., Zorea J., Elkabets M., Wolfson M., Fraifeld V.E. (2019). c-Met as a new marker of cellular senescence. Aging.

[45] Mikuła-Pietrasik J., Uruski P., Pakuła M., Maksin K., Szubert S., Woźniak A., Naumowicz E., Szpurek D., Tykarski A., Książek K. (2017). Oxidative stress contributes to hepatocyte growth factor-dependent pro-senescence activity of ovarian cancer cells. Free Radic. Biol. Med..

[46] Sreeja S.R., Lee H.Y., Kwon M., Shivappa N., Hebert J.R., Kim M.K. (2019). Dietary Inflammatory Index and Its Relationship with Cervical Carcinogenesis Risk in Korean Women: A Case-Control Study. Cancers.

[47] Rayburn E.R., Wang W., Zhang Z., Li M., Zhang R., Wang H. (2006). Experimental therapy of prostate cancer with an immunomodulatory oligonucleotide: Effects on tumor growth, apoptosis, proliferation, and potentiation of chemotherapy. Prostate.

[48] Hickson L.J., Langhi Prata L.G.P., Bobart S.A., Evans T.K., Giorgadze N., Hashmi S.K., Herrmann S.M., Jensen M.D., Jia Q., Jordan K.L. (2019). Senolytics decrease senescent cells in humans: Preliminary report from a clinical trial of Dasatinib plus Quercetin in individuals with diabetic kidney disease. EBioMedicine.

[49] Purohit S., Sharma A., Hopkins D., Steed L., Bode B., Anderson S.W., Reed J.C., Steed R.D., Yang T., She J.X. (2015). Large-Scale Discovery and Validation Studies Demonstrate Significant Reductions in Circulating Levels of IL8, IL-1Ra, MCP-1, and MIP-1β in Patients with Type 1 Diabetes. J. Clin. Endocrinol. Metab..

[50] Friedman J.H., Hastie T., Tibshirani R. (2010). Regularization Paths for Generalized Linear Models via Coordinate Descent. J. Stat. Softw..

